# Socio-demographic, Clinical, and Genetic Determinants of Quality of Life in Lung Cancer Patients

**DOI:** 10.1038/s41598-018-25712-1

**Published:** 2018-07-13

**Authors:** Jeanne A. Pierzynski, Yuanqing Ye, Scott M. Lippman, Maria A. Rodriguez, Xifeng Wu, Michelle A. T. Hildebrandt

**Affiliations:** 1 Department of Epidemiology, The University of Texas MD Anderson Cancer Center, Houston, Texas USA; 2 Department of Medicine, University of California at San Diego Moores Cancer Center, La Jolla, California USA; 3 Department of Lymphoma/Myeloma, Division of Cancer Medicine, The University of Texas MD Anderson Cancer Center, Houston, Texas USA

## Abstract

Patient reported health-related quality of life (QOL) is a major component of the overall well-being of cancer patients, with links to prognosis. In 6,420 lung cancer patients, we identified patient characteristics and genetic determinants of QOL. Patient responses from the SF-12 questionnaire was used to calculate normalized Physical Component Summary (PCS) and Mental Component Summary (MCS) scores. Further, we analyzed 218 single nucleotide polymorphisms (SNPs) in the p38 MAPK signaling pathway, a key mediator of response to cellular and environmental stress, as genetic determinants of QOL in a subset of the study population (N = 641). Trends among demographic factors for mean PCS and MCS included smoking status (PCS P_trend_ < 0.001, MCS P_trend_ < 0.001) and education (PCS P_trend_ < 0.001, MCS P_trend_ < 0.001). Similar relationships were seen for MCS. The homozygous rare genotype of *MEF2B*: rs2040562 showed an increased risk of a poor MCS (OR: 3.06, 95% CI: 1.05–8.92, P = 0.041). Finally, survival analysis showed that a low PCS or a MCS was associated with increased risks of five-year mortality (HR = 1.63, 95% CI: 1.51–1.77, HR = 1.23, 95% CI: 1.16–1.32, respectively) and there was a significant reduction in median survival time (P_log-rank_ < 0.001). These findings suggest that multiple factors contribute to QOL in lung cancer patients, and baseline QOL can impact survival.

## Introduction

Newly diagnosed lung cancer patients experience one of the worst symptom burdens^1^. In recent years, health-related quality of life (QOL) has become an important aspect of cancer treatment and research has linked improved patient-reported QOL to improved lung cancer survival^2,3^.

To date, several studies have investigated the role of demographic factors on QOL in cancer patients. African American men recently diagnosed with prostate cancer and African American women breast cancer survivors reported better emotional well-being compared to Caucasians^4,5^. Older age has been shown to be a predictor of emotional and physical well-being for multiple cancer sites^6^. Previous studies suggested that women with lung cancer report higher rates of depression prior to treatment (49%) than men (29%), and depression is a strong indicator of QOL^7,8^. In small cell lung cancer (SCLC) patients, one study showed that those smoking one year post diagnosis exhibited the worst QOL compared to all other smoking categories^9^, while another study reported inconsistent findings with those who continue to smoke following diagnosis reporting worse QOL^10^. Clinical factors may also play a role in QOL. SCLC patients reported worse depression and anxiety than non-small cell lung cancer (NSCLC) patients^7^. While evidence suggests that demographic and clinical characteristics are predictors of cancer patient QOL, inter-individual variability still remains.

Genetic components may also affect QOL. For example, one study reported an association between three SNPs in two genes related to inflammation (*LTA* and *PTGS2*) and pain severity, social functioning, and mental health in lung cancer survivors^11^. The p38 MAPK pathway is activated through extracellular stimuli such as proinflammatory cytokines including interleukin (IL)-1) and tumor necrosis factor (TNF) alpha^12^. Once the p38 MAPK pathway is activated, the downstream effects ultimately result in changes in cell survival through programmed cell death^13^ and pathway activation can lead to the increased production of more pro-inflammatory cytokines^14^. This pathway is of interest in regards to QOL because it is a key mediator of response to cellular and environmental stress. Examples of stress that activate this pathway are pro-inflammatory cytokines (as stated above, such as IL-1 and TNF-alpha). Further, because this can result in the production of more pro-inflammatory cytokines, this is of interest because elevated levels of pro-inflammatory cytokines have been associated with negative symptoms in cancer patients (such as fatigue and depression) of which can negatively impact QOL^15^. To date, no study has examined genetic variation in this pathway in relation to QOL in lung cancer patients. In this study, we assessed the relationship between QOL and lung cancer survival and identified determinants of QOL by investigating the relationship between patient characteristics and genetic factors.

## Results

### Host Characteristics

The patient characteristics are shown in Table 1. The mean age was 60.9 years. Most patients were whites (83%), married (72.9%), and had completed at least a high school education (57.7%), 17.4% were never smokers, 41.2% were never alcohol consumers, and 27.5% of the patients were diagnosed with stage IV disease. The distribution of PCS and MCS scores in the study population show that neither score was normally distributed, Supplemental Fig. 1.

**Table 1:** Host Characteristics and the Association of Demographic, Lifestyle, and Clinical Characteristics with PCS and MCS Score.

Characteristic	N	%	PCS, Mean (SD)	P value	MCS, Mean (SD)	P value
**Age**						
<50	1,081	16.84	39.05 (11.60)		45.37 (10.89)	
50-59	1,672	26.04	39.12 (11.95)	1.000	44.57 (11.33)	0.351
60-69	2,149	33.47	38.50 (11.93)	0.759	46.23 (11.35)	0.221
70+	1,518	23.64	38.44 (11.59)	0.715	47.48 (11.45)	<0.001
**P for trend**				0.077		<0.001
**Sex**						
Male	3,431	53.46	38.96 (11.90)		46.75 (11.33)	
Female	2,987	46.54	38.49 (11.69)	0.109	45.03 (11.29)	<0.001
**Marital status**						
Married	4,679	72.93	39.24 (11.75)		46.33 (11.19)	
Widowed	635	9.90	37.00 (11.73)	<0.001	45.83 (11.84)	0.972
Separated	34	0.53	36.06 (11.91)	0.710	42.19 (11.27)	0.290
Divorced	637	9.93	37.24 (11.64)	0.001	44.13 (11.66)	<0.001
Never Married	431	6.72	38.23 (12.30)	0.606	44.96 (11.37)	0.154
**Education**						
<High School	760	12.37	35.17 (11.22)		42.63 (11.81)	
High School/Vocational/AA	3,543	57.66	38.02 (11.60)	<0.001	45.85 (11.45)	<0.001
College Degree	1,842	29.98	41.77 (11.71)	<0.001	47.64 (10.59)	<0.001
**P for trend**				<0.001		<0.001
**Race**						
Non-Hispanic White	5,268	83.09	38.92 (11.75)		45.97 (11.34)	
Hispanic	280	4.42	39.05 (11.73)	1.000	44.56 (11.71)	0.355
Non-Hispanic Black	460	7.26	35.73 (11.97)	<0.001	45.21 (11.40)	0.849
Asian/Pacific Islander	217	3.42	40.62 (11.54)	0.316	48.26 (10.68)	0.034
Other	115	1.81	37.80 (12.21)	0.976	47.27 (10.90)	0.918
**Alcohol**						
Never	2,626	41.19	37.19 (11.50)		45.48 (11.58)	
Former	1,205	18.90	35.37 (11.21)	<0.001	44.48 (11.58)	0.033
Current	2,544	39.91	41.93 (11.64)	<0.001	47.14 (10.86)	<0.001
**P for trend**				<0.001		<0.001
**Smoking**						
Never	1,112	17.40	40.78 (11.86)		48.02 (10.51)	
Former	4,083	63.88	38.85 (11.73)	<0.001	46.06 (11.29)	<0.001
Current	1,197	18.73	36.47 (11.65)	<0.001	43.61 (11.88)	<0.001
**P for trend**				<0.001		<0.001
**Past Medical History**						
Yes	5,394	84.43	38.37 (11.78)		45.81 (11.45)	
No	995	15.57	40.61 (11.80)	<0.001	46.73 (10.66)	0.018
**Past Cancer Treatment**						
Yes	1,845	28.74	35.86 (10.62)		45.39 (11.15)	
No	4,575	71.26	39.90 (12.06)	<0.001	46.17 (11.41)	0.012
**Histology**						
**Non-Small Cell Carcinoma**						
Adenocarcinoma	2,996	49.32	39.66 (11.82)		46.63 (11.08)	
Squamous Cell	973	16.02	38.03 (11.46)	0.003	45.69 (11.73)	0.305
Large Cell	208	3.42	37.36 (11.33)	0.091	45.00 (11.30)	0.500
Non-small cell carcinoma, non-specified	972	16.00	37.40 (11.85)	<0.001	45.20 (11.38)	0.009
**Small Cell**	667	10.98	36.84 (11.67)	<0.001	43.77 (11.62)	<0.001
**Other**	258	4.25	41.51 (11.92)	0.200	47.65 (11.37)	0.930
**Stage**						
I	621	9.67	43.90 (11.46)		49.28 (10.39)	
II	228	3.55	43.68 (11.74)	1.000	50.09 (10.37)	0.988
III	979	15.25	41.16 (11.79)	<0.001	46.26 (11.21)	<0.001
IV	1,768	27.54	37.74 (11.82)	<0.001	45.22 (11.52)	<0.001
Unknown	2,824	43.99	37.00 (11.30)	<0.001	45.23 (11.34)	<0.001
**P for trend**				<0.001		<0.001

### Relationship between Demographic and Clinical Characteristics and PCS/MCS Scores

#### Comparison of Mean PCS Scores

Table 1 shows patients who had a college degree (41.8, P < 0.001) reported a higher mean PCS score when compared to individuals with less than a high school education (35.2). Interestingly, current drinkers had a higher mean PCS (41.9, P < 0.001) score compared to never drinkers (37.2). The opposite effect was seen for current smokers (36.5, P < 0.001) when compared to never smokers (40.8). When examining racial differences, blacks were more likely to have a low mean PCS (35.7, P < 0.001) score compared to whites (38.9). The mean PCS score for widowed patients (37.0, P < 0.001) was lower compared to those married (39.2). The PCS scores for patients with squamous cell (38.0, P = 0.003) and small cell (36.8, P < 0.001) lung cancer were lower than those with adenocarcinoma (39.7). Finally, stage III (41.2, P < 0.001) and stage IV (37.7, P < 0.001) lung cancer patients reported worse PCS scores compared to those with stage I (43.9).

#### Poor PCS Risk

**Figure 1:**
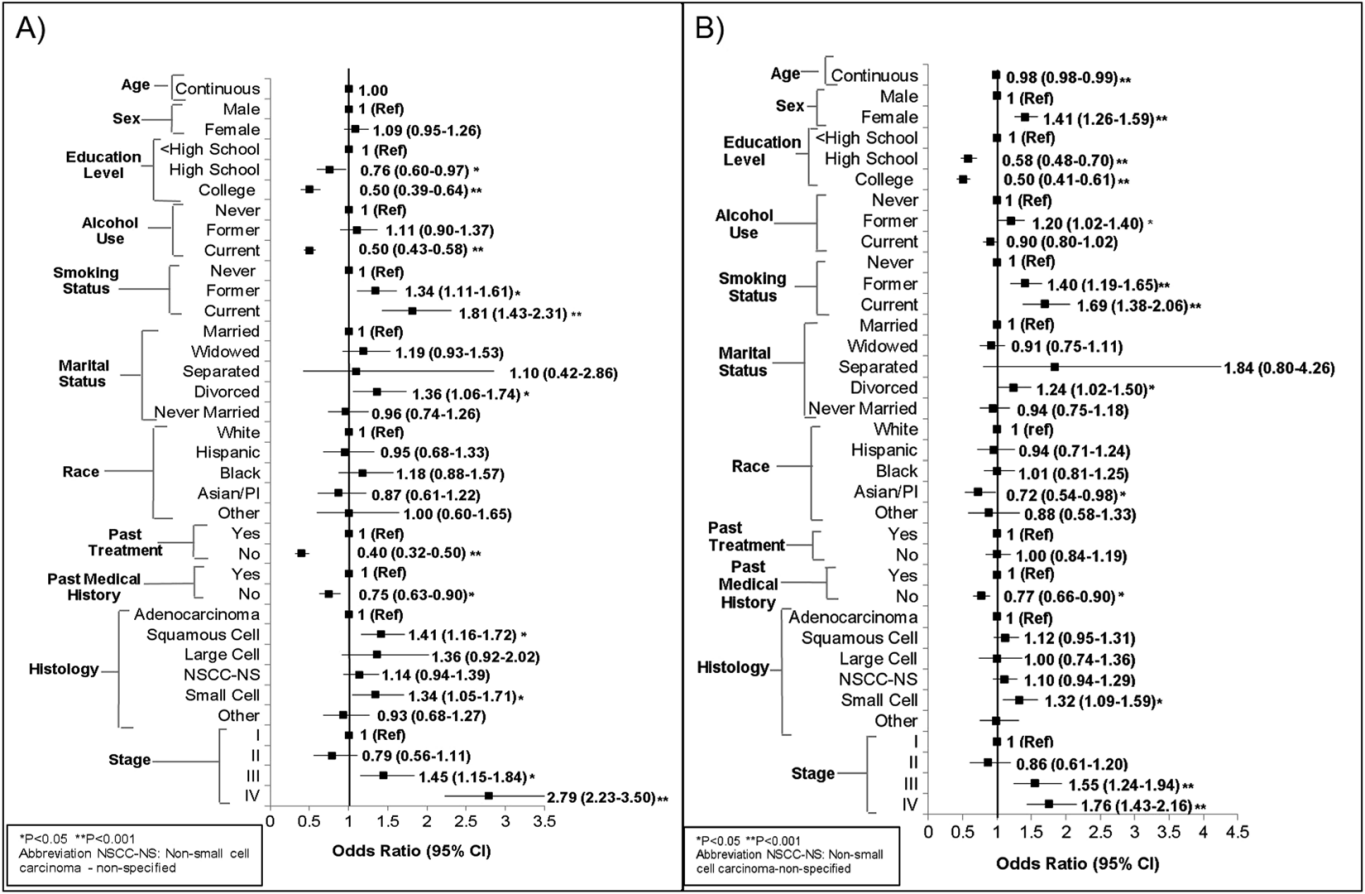
Association between Demographic/Clinical Factors and Quality of Life Measures in Lung Cancer Patients; (**A**) PCS scores, (**B**) MCS scores. Odds ratios adjusted by age, sex, race, marital status, education, smoking status, alcohol use, past medical treatment, past treatment, histology, and stage.

We examined the factors contributing to poor PCS risk as shown in Fig. 1A (Supplemental Table 1). When compared to less than a high school education, patients with a college degree had a lower risk of reporting a low PCS (OR = 0.50, 95% CI: 0.39–0.64, P < 0.001). When compared to never smokers, former smokers had a higher risk of reporting a poor PCS (OR = 1.34, 95% CI: 1.11–1.61, P = 0.002). Current smokers (compared to never smokers) had an even larger increased risk of reporting a poor PCS (OR = 1.81, 95% CI: 1.43–2.31, P < 0.001). Squamous cell lung cancer patients were at a 41% increased risk of a poor PCS (OR = 1.41, 95% CI: 1.16–1.72, P = 0.001). Individuals diagnosed with stage III (OR = 1.45, 95% CI: 1.15–1.84, P = 0.002) and IV (OR = 2.79, 95% CI: 2.23–3.50, P < 0.001) lung cancer were at an increased risk of an unfavorable PCS score.

#### Comparison of Mean MCS Score

Table 1 showed as participant’s age increased, their MCS score increased and the oldest age group reported a higher mean MCS score (70+: 47.5, P < 0.001) compared to the youngest age group (<50: 45.4), indicating their perception of their QOL was better than the youngest age group. MCS scores for patients who had a high school, vocational, or associates degrees (45.9, P < 0.001) or a college degree (47.6, P < 0.001) were higher compared to those who did not finish high school (42.6, P_trend_ < 0.001). Asian/Pacific Islanders (48.3, P = 0.034) reported higher mean MCS scores compared to whites (46.0). The lowest MCS score for alcohol usage was seen with former alcohol drinkers (44.5, P = 0.033) and was worse compared to never drinkers (45.5). Interestingly, current drinkers (47.1, P < 0.001) reported a higher MCS score compared to never drinkers. A downward trend of mean MCS scores was see for former (46.1, P < 0.001) and current (43.6, P < 0.001) smokers compared to never smokers (48.0, P_trend_ < 0.001). Divorced patients were more likely to have a worse mean MCS score (44.1, P < 0.001) compared to married patients (46.3). By histology type, patients with small cell lung cancer had the lowest MCS score (43.8, P < 0.001) compared to those with adenocarcinoma (46.6). When stratifying by smoking, this relationship was only seen in ever smokers (data not shown). Finally, stage III (46.3, P < 0.001) or IV (45.2, P < 0.001) patients reported worse MCS scores compared to those with stage I (49.3) lung cancer.

#### Poor MCS Risk

We examined the factors contributing to poor MCS shown in Fig. 1B (Supplemental Table 2). Females were 41% (OR = 1.41, 95% CI: 1.26–1.59, P < 0.001) more likely to report a worse MCS when compared to males. Participants with a college degree had a lower risk of reporting a low MCS compared to patients with less than a high school education (OR = 0.50, 95% CI: 0.41–0.61, P < 0.001). Asian/Pacific Islanders were more likely to report a better MCS (OR = 0.72, 95% CI: 0.54–0.98, P = 0.035) compared to whites. When compared to never smokers, former smokers had a higher risk of reporting a poor MCS (OR = 1.40, 95% CI: 1.19–1.65, P < 0.001). Current smokers (compared to never smokers) had an even bigger increased risk of reporting a poor MCS (OR = 1.69, 95% CI: 1.38–2.06, P < 0.001). Finally, patients diagnosed with stage IV lung cancer have a 76% (OR = 1.76, 95% CI: 1.43–2.16, P < 0.001) greater risk of reporting a worse MCS score compared to those diagnosed with stage I.

### The Relationship between Genetic Variants in the p38 MAPK Pathway and PCS/MCS Scores

#### Discovery Phase for PCS and MCS Scores

In the discovery phase, 29 SNPs were associated with PCS score (Supplemental Table 3) and 20 SNPS were associated with MCS score (Supplemental Table 4). The most significant genetic variant associated with PCS score was *TNFRSF1B*: rs496888, which was associated with a higher PCS score (OR = 0.40, 95% CI: 0.21–0.75, P = 0.004) under the dominant model. The most significant variant associated with MCS score was located in *MAP2K3 (*rs1466314) under the dominant model, with patients showing an over 2-fold increased risk of a poor MCS score (OR: 2.25, 95% CI: 1.31–3.87, P = 0.003).

When analyzing the gene-based analysis results from the VEGAS software, many genes were significant contributors to PCS and MCS scores in the discovery phase (data not shown). For PCS score this included *MAPK11* (P = 0.011) and *PEX7* (P = 0.005). For MCS score, this included *MAP2K3* (P = 0.002) and *TRAF2* (P = 0.023).

In the discovery phase, individuals with homozygous variant genotype of *MEF2B*: rs2040562 showed a 3.06-fold increased risk of a poor mental health score (95% CI: 1.05–8.92, P = 0.041), compared to subjects carrying at least one major allele. Individuals with homozygous variant genotype of *MEF2B*: rs2040562 showed a 2.61-fold increased risk of a poor MCS score (95% CI: 1.11–6.15, P = 0.028) in the validation phase. When we combined discovery and validation phase: OR = 2.43, 95% CI: 1.29–4.58, P = 0.006 for rare homozygote genotype (Table 2). When analyzing the gene-based analysis results, *MAP2K6* was a contributor to PCS score based on the discovery phase p-values (P = 0.022) and the validation phase p-values (P = 0.001) (data not shown).

**Table 2:** Association Between p38 MAPK Validated Variant and MCS Score.

Gene: SNP	Model	MCS <50 WW/WV/VV	MCS ≥50 WW/WV/VV	OR (95% CI)*	P Value
**Discovery Phase**					
*MEF2B*: rs2040562	Recessive	86/60/23	75/70/7	3.06 (1.05–8.92)	0.041
**Validation Phase**					
**Gene: SNP**	**Model**	**MCS** <**50 WW/WV/VV**	**MCS ≥50 WW/WV/VV**	OR (95% CI)*	**P Value**
*MEF2B:* rs2040562	Recessive	62/76/28	56/84/14	2.61 (1.11–6.15)	0.028
**Combined Analysis**					
**Gene: SNP**	**Model**	**MCS** <**50 WW/WV/VV**	**MCS ≥50 WW/WV/VV**	**OR (95% CI)**	**P Value**
*MEF2B*: rs2040562	Recessive	148/136/51	131/154/21	2.43 (1.29–4.58)	0.006

*Adjusted for age, sex, marital status, education, smoking status, alcohol use, past medical history, past treatment, histology, and stage.

#### Relationship between PCS/MCS Scores, SNPs, and Survival

**Figure 2:**
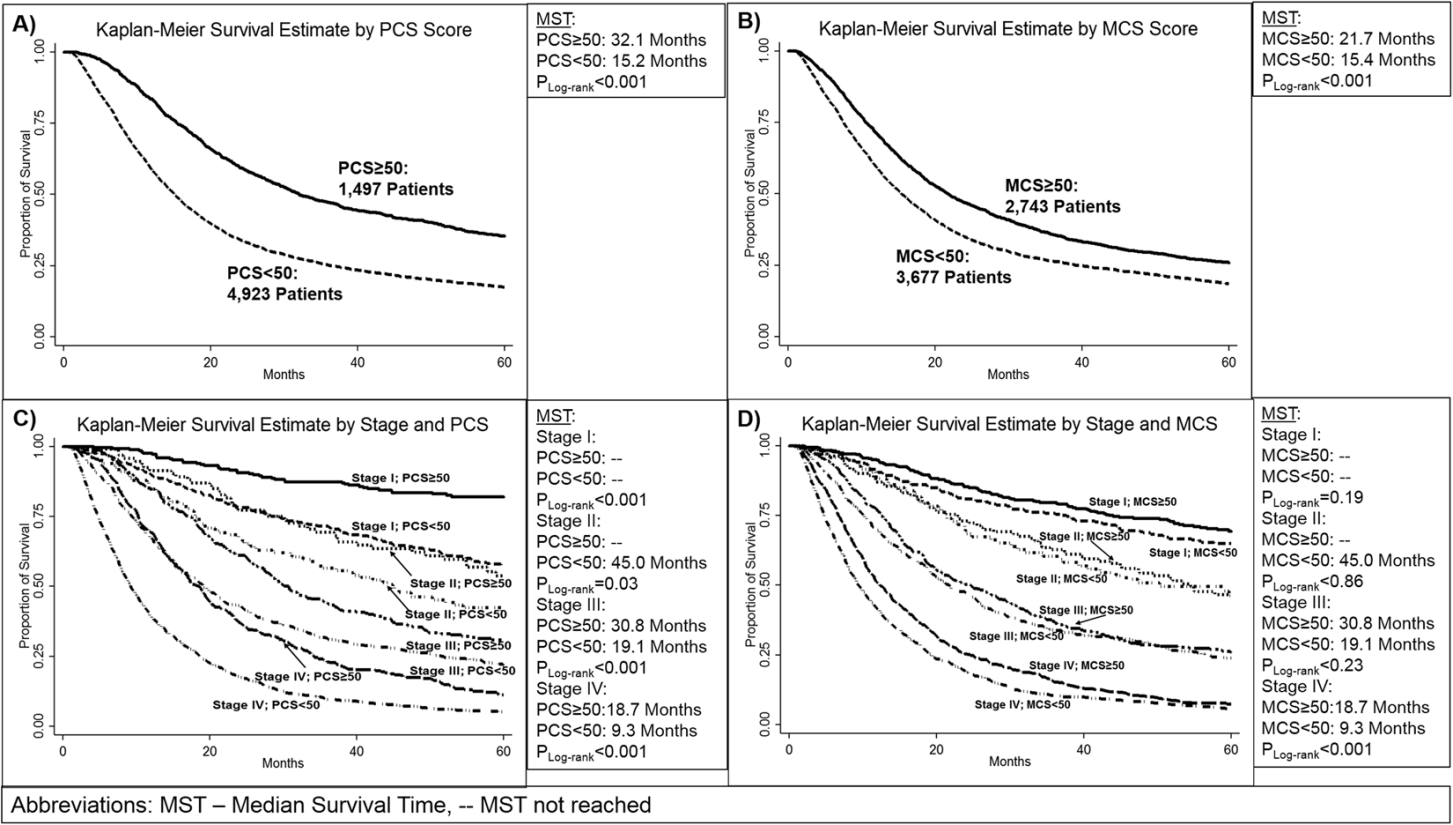
5-year Survival by Quality of Life Measures in Lung Cancer Patients and Cancer Stage; (**A**) PCS scores (**B**) MCS scores; Scores were dichotomized at 50, representing the mean PCS/MCS score in the general population. Scores were dichotomized at 50 and stratified by cancer stage. Hazard Ratios adjusted by age, sex, race, smoking status, previous cancer treatment, treatment at MD Anderson, histology, and stage.

Survival analysis in Fig. 2 and Supplemental Table 5 showed that individuals with a PCS or MCS score less than 50, had an increased risk of death (PCS: HR = 1.63, 95% CI: 1.51–1.77, P < 0.001, MCS: HR = 1.23, 95% CI: 1.16–1.32, P < 0.001). This increased risk resulted in a difference between median survival time (MST) of those with a PCS score less than 50 (MST = 15.1 months) and those with a PCS score greater than 50 (MST = 32.1 months, P_log-rank_ < 0.001) (Figure 2A). There was also a reduction in MST for patients with a MCS score less than 50 at only 15.4 months and those with a MCS greater than 50 at 21.7 months (P_log-rank_ < 0.001) (Fig. 2B). When stratifying by stage, this effect was seen in stage III and stage IV patients (Figure 2C and D).

We found that eighteen SNPs were significantly associated with lung cancer five-year survival in the discovery phase (Supplemental Table 6). Patients with this *MAP3K5*: rs3765259 variant had a decreased risk of dying (HR = 0.56, 95% CI: 0.40–0.79, P = 0.0008). Those with the common genotype had a MST of 17.3 months, those with one variant allele had a MST of 23.8 months (P_log-rank_ = 0.053).

## Discussion

The importance of a good QOL in cancer patients is well known. To date there have been no studies reported on the association of detailed demographic, clinical characteristics, and p38 MAPK genetic variants and QOL in lung cancer patients. This study characterizes the epidemiological, clinical, and genetic determinants of QOL in a large population of lung cancer patients. Alcohol use, smoking, education, and higher lung cancer stage were consistently shown to impact mean PCS and MCS. Poor PCS and MCS QOL scores were associated with and increased risk of death and poor survival. A validated SNP (*MEF2B*: rs2040562) in the p38 MAPK pathway was associated with an increased risk of poor MCS score.

Previous research has found that the majority of smokers who are current smokers when diagnosed with lung cancer will continue to smoke regardless of their cancer diagnosis^16^. In our study, former and current smokers reported worse PCS and MCS scores compared to never smokers, and poor QOL in current smokers is consistent with the literature^9,10^. Former smokers reported slightly higher PCS and MCS scores than current smokers. Suggesting that participants who are former smokers do not feel that their QOL is as high as never smokers. This presents the possibility of future smoking cessation programs in lung cancer survivors to assist current smokers in becoming former smokers with the goal of increasing their QOL and thus, potentially improving long-term prognosis.

A previous study examining health perceptions in lung cancer survivors found that current drinkers at diagnosis will continue to be current drinkers^17^. Those that were currently drinking were at a higher risk of reporting worse perception of health status^17^. Interestingly, we found the opposite finding in our analysis for physical QOL in newly diagnosed patients. Our analysis is the first to our knowledge that has examined alcohol use and QOL in newly diagnosed lung cancer cases. Further analysis is warranted to dissect the potentially complicated relationship between alcohol use and QOL.

There is limited research on the association between education level and QOL in lung cancer patients. A few studies found that lower education level is associated with poorer performance status in clinical trial participants^18,19^ and higher education is associated with better QOL and lower symptom levels^20^. Mixed results have been seen between education level and different aspects of QOL in NSCLC patients^21^ and survivors^22^. Our study is the first to examine education level and QOL in a large population of newly diagnosed patients and we found patients with a high school degree or higher were more likely to report higher PCS and MCS scores. Further research is needed to determine possible disparities underlying the gap between QOL and education level in patients.

The p38 MAPK pathway has been associated with QOL and QOL factors such as depression, pain, and there is evidence of an association with anxiety in animal models^23–25^. Individuals diagnosed with major depression have increased levels of pro-inflammatory cytokines and corresponding receptors in peripheral blood and cerebral spinal fluid^25,26^, and pro-inflammatory cytokines activate the p38 MAPK pathway, which subsequently can activate the serotonin transporter (SERT)^27^. Furthermore, research has linked the activation of the p38 MAPK pathway to regulation of mood-related neurotransmitters, with potential links to the regulation of synaptic plasticity^28^. Our study discovered multiple variants in p38 MAPK pathway genes that were associated with PCS and MCS scores. One variant (*MEF2B*: rs2040562) was replicated in association with mental QOL. Myocyte-enhancing factor 2B (MEF2B) protein is a transcription factor that is important in development and adulthood and is important in regulating transcriptional programming^29^. Research has shown that patients with metastatic renal cell carcinoma who are depressed (compared to non-depressed patients) show increased expression of *MEF2*^30^. Our results suggest that individuals with a variant in *MEF2B* are at an increased risk of reporting a poor MCS score and further research should be completed to understand the mechanism.

Finally, we identified that individuals with poor reported PCS or MCS scores are at a higher risk of five-year mortality and our results support and extend previous findings^31–35^ that examined QOL during or following treatment. We examined QOL at time of diagnosis and studies that examined baseline QOL and survival in lung cancer patients support our findings^2,3,36–42^. These results further highlight that many factors influence survival and stress the importance of potential behavioral interventions in the clinical setting to improve QOL and potentially improve survival.

The strengths of this study include a large study population and the ability to assess the relationship of various demographic, epidemiological, clinical, and genetic factors with QOL. The main limitation of this study is that over 2,800 patients were missing stage information. The results of a sensitivity analysis showed consistency between the full model and the reduced model.

In conclusion, we have identified several determinants that contribute to QOL in lung cancer patients. The results of this study are important in that they provide an overarching picture of key QOL factors that affect lung cancer patients. This information could be used to identify potential interventions to improve QOL, as well as those at increased risk of a poor treatment response and prognosis due to their reduced QOL. This could result in more of a proactive approach in the clinic to address health behaviors that impact QOL.

## Materials and Methods

### Study Population and Data Collection

The population was 6420 newly diagnosed lung cancer patients from The University of Texas MD Anderson Cancer Center collected from 2000 to 2010. Participants completed an institutional patient health intake questionnaire at their initial visit to MD Anderson within one year of diagnosis. Since 1999, the SF-12v1 has been part of MD Anderson’s institutional patient intake questionnaire completed by all new patients at MD Anderson Cancer Center, which also includes demographic and epidemiological data. It encompasses four domains of QOL (physical, social, functional, and emotional) and eight subscales (physical functioning, general health, bodily pain, role physical, vitality, social functioning, role emotional, and mental health) formed from the SF-12v1 responses^43^. These subscales are used to calculate the Physical Component Summary (PCS) and Mental Component Summary (MCS) scores. Both the MSC and PCS were normalized to a mean of 50 (SD = 10) based on responses to the SF-12v1 among the US general population. A score greater than 50 indicates a QOL that is better than the general population. The question in the SF-12v1 that asks “During the past 4 weeks, how much did pain interfere with your normal work” was modified in the questionnaire as “During the past week, has pain interfered with your general activities” and the scoring was adjusted to match the SF-12v1 scoring. Current alcohol drinkers were participants that self-report drinking at least one alcoholic drink per month. Never smokers were participants who had smoked less than 100 cigarettes in their lifetime. Clinical data were obtained from MD Anderson’s Tumor Registry. Individuals with multiple primary tumors were excluded, except for multiple lung tumors. The participants provided written informed consent and the study was approved by the Institutional Review Board at The University of Texas MD Anderson Cancer Center. All methods were performed in accordance with the relevant guidelines and regulations.

### DNA Isolation and Genotyping

A subset of patients (N = 641) have data on 218 SNPS in 20 genes from the p38 MAPK pathway. DNA isolation and genotyping methods have been previously described^44,45^. In short, inflammation pathway-related genes were identified through the Gene Oncology database^46,47^ and the National Center for Biotechnology Information (NCBI) Pubmed^48^. Haplotype tagging SNPs were selected for each gene 10 kb upstream of the transcriptional start site or 10 kb downstream of the transcriptional stop site. SNPs in the coding (nonsynonymous SNPs and synonymous SNPs) and regulatory regions (splicing site, promoter, 5′UTR, and 3′UTR). In addition, SNPs previously reported to be associated with cancer and functional SNPs were included. Genotyping was completed using the Illumina Infinium iSelect HD Custom Genotyping BeadChip.

### Statistical Analysis

To analyze the difference in mean PCS and MCS scores between categories of host characteristics, t-test or ANOVA with pairwise comparison testing was used (SIDAK test). Non-parametric tests were completed as well and the results were similar. The parametric results are reported. PCS and MCS were dichotomized by 50 to assess the association of demographic and clinical variables with QOL. Unconditional multivariable logistic regression was used to calculate odd ratios (ORs) and 95% confidence intervals (CIs). Confounders were adjusted for in the PCS and MCS multivariable models (age, sex, marital status, education, smoking status, alcohol use, past medical history, past treatment, histology, and stage). A sensitivity analysis was completed and found no major differences between the full data (missing stage categorized as unknown) and the reduced data (missing stage removed) (Supplemental Figs 3 and 4). The full dataset was used. For the effect of 218 SNPs in the p38 MAPK pathway on PCS and MCS risk, unconditional multivariable logistic regression was used to estimate ORs and 95% Cis (age, sex, marital status, education, smoking status, alcohol use, past medical history, past treatment, histology, and stage). Dominant, recessive, and additive models of inheritance were assessed for each SNP. The study sample was divided into two groups by assigning alternating samples into the discovery set and the validation set. Q-Values were also calculated to test for multiple comparisons in the discovery phase (data not shown). We tested all of the variants from the discovery phase that had a p-value of less than 0.05 in the validation phase of the genetic analysis. Multivariable Cox regression was used to estimate hazard ratios (HR) and 95% CI for all survival analyses (adjusted for age, sex, race, smoking status, previous cancer treatment, treatment at MD Anderson, histology, and cancer stage). The effects of PCS and MCS scores on five-year lung cancer survival (calculated using the diagnosis date and last contact date) were estimated. Kaplan-Meier survival curves and log-rank tests were calculated to analyze the difference in five-year survival times. Statistics were completed using STATA 13 (Stata Corporation, College Station, TX). Statistical tests were two sided and a p-value of less than 0.05 was considered significant. VEGAS software was used to perform a gene-based interaction analysis^49^. In the VEGAS analysis, significant variants were carried forward and the validation set was analyzed using the same model that was most significant in the discovery phase.

### Data availability

The datasets generated during and/or analyzed during the current study are available from the corresponding author on reasonable request.

## Electronic supplementary material

Supplemental Figure and Tables
