# Supplementary material for: Socio-demographic, Clinical, and Genetic Determinants of Quality of Life in Lung Cancer Patients
Source: Sci Rep. 2018 Jul 13;8:10640. doi: 10.1038/s41598-018-25712-1 (PMC6045646; doi:10.1038/s41598-018-25712-1)
Supplement: Supplementary file 1 — Supplemental Figure and Tables [file 41598_2018_25712_MOESM1_ESM.doc]

**Socio-demographic, Clinical, and Genetic Determinants of Quality of Life in Lung Cancer Patients**

**Jeanne A. Pierzynski MPH^1^, Yuanqing Ye PhD^1^, Scott M. Lippman MD^2^, Maria A. Rodriguez MD^3^, Xifeng Wu MD PhD^1^, Michelle A.T. Hildebrandt PhD^1^**

**Supplemental Figure and Tables**

**
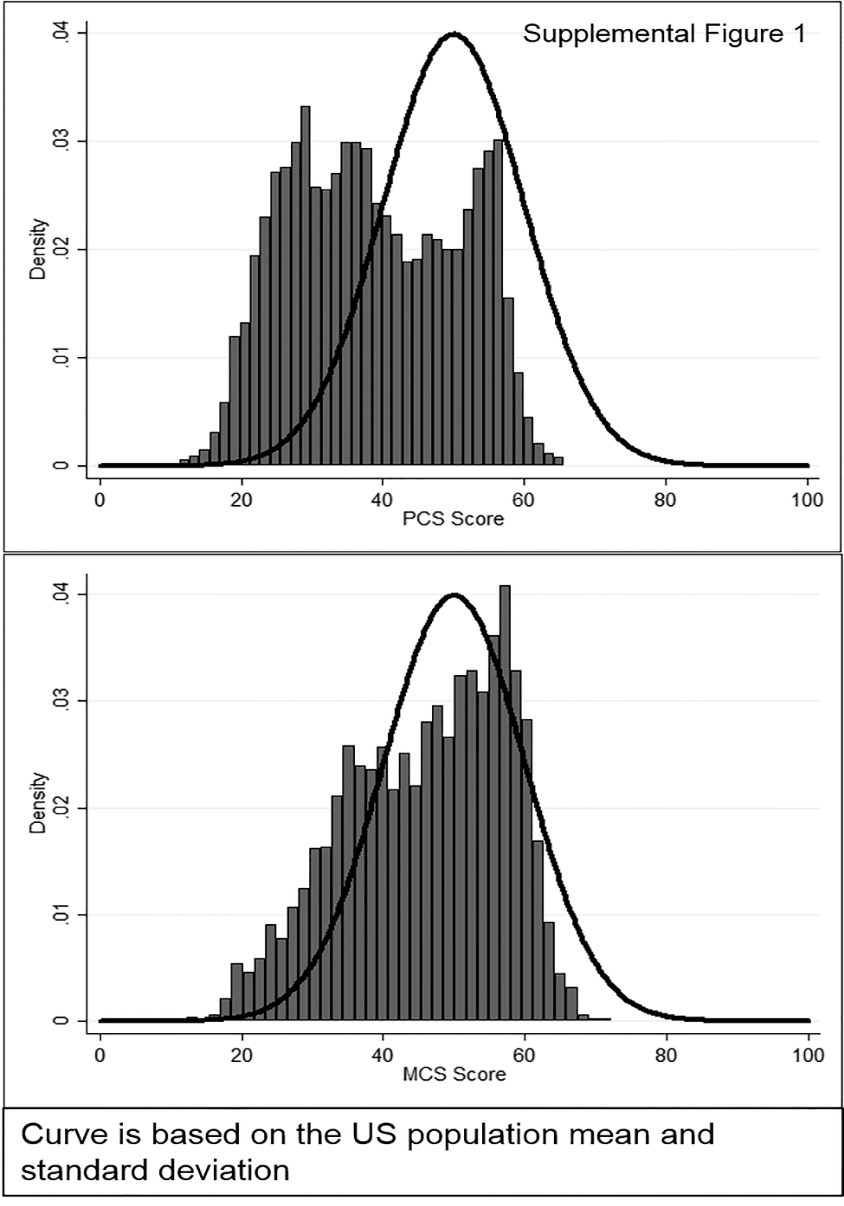
**

**Supplemental Figure 1. Distribution of PCS and MCS QOL scores in the study population compared to the distribution in the US general population**

| **Supplemental Table 1. Association of Host Characteristics and PCS Scores** | | | | | |
| --- | --- | --- | --- | --- | --- |
| **Variable** | **PCS<50** | **PCS≥50** | **OR** | **95% CI** | **P Value** |
| Age (Continuous) | 4376 | 1329 | 1.00 | 1.00-1.01 | 0.246 |
| **Sex** |  |  |  |  |  |
| Male | 2295 | 735 | 1.00 (Ref) |  |  |
| Female | 2081 | 594 | 1.09 | 0.95-1.26 | 0.215 |
| **Education** |  |  |  |  |  |
| < High School | 596 | 102 | 1.00 (Ref) |  |  |
| High School/Vocational/AA | 2603 | 687 | 0.76 | 0.60-0.97 | 0.026 |
| College Degree | 1177 | 540 | 0.50 | 0.39-0.64 | <0.0001 |
| **Alcohol Use** |  |  |  |  |  |
| Never | 1914 | 423 | 1.00 (Ref) |  |  |
| Former | 909 | 159 | 1.11 | 0.90-1.37 | 0.268 |
| Current | 1553 | 747 | 0.50 | 0.43-0.58 | <0.0001 |
| **Smoking Status** |  |  |  |  |  |
| Never | 690 | 298 | 1.00 (Ref) |  |  |
| Former | 2813 | 839 | 1.34 | 1.11-1.61 | 0.002 |
| Current | 873 | 192 | 1.81 | 1.43-2.31 | <0.0001 |
| **Past Medical History** |  |  |  |  |  |
| Yes | 3733 | 1080 | 1.00 (Ref) |  |  |
| No | 643 | 249 | 0.75 | 0.63-0.90 | 0.002 |
| **Marital Status** |  |  |  |  |  |
| Married | 3164 | 1026 | 1.00 (Ref) |  |  |
| Widowed | 453 | 109 | 1.19 | 0.93-1.53 | 0.166 |
| Separated | 24 | 6 | 1.10 | 0.42-2.86 | 0.850 |
| Divorced | 455 | 95 | 1.36 | 1.06-1.74 | 0.014 |
| Never Married | 280 | 93 | 0.96 | 0.74-1.26 | 0.787 |
| **Race** |  |  |  |  |  |
| White | 3631 | 1127 | 1.00 (Ref) |  |  |
| Hispanic | 178 | 55 | 0.95 | 0.68-1.33 | 0.763 |
| Black | 346 | 68 | 1.18 | 0.88-1.57 | 0.268 |
| Asian/Pacific Islander | 145 | 56 | 0.87 | 0.61-1.22 | 0.409 |
| Other | 76 | 23 | 1.00 | 0.60-1.65 | 0.995 |
| **Past Treatment** |  |  |  |  |  |
| Yes | 1400 | 211 | 1.00 (Ref) |  |  |
| No | 2976 | 1118 | 0.40 | 0.32-0.50 | <0.0001 |
| **Histology** |  |  |  |  |  |
| **Non-Small Cell** |  |  |  |  |  |
| Adenocarcinoma | 2093 | 731 | 1.00 (Ref) |  |  |
| Squamous Cell | 720 | 190 | 1.41 | 1.16-1.72 | 0.001 |
| Large Cell | 155 | 36 | 1.36 | 0.92-2.02 | 0.122 |
| Non-small cell carcinoma – non-specified | 727 | 186 | 1.14 | 0.94-1.39 | 0.178 |
| **Small Cell** | 519 | 105 | 1.34 | 1.05-1.71 | 0.018 |
| **Other** | 162 | 81 | 0.93 | 0.68-1.27 | 0.641 |
| **Stage** |  |  |  |  |  |
| I | 338 | 226 | 1.00 (Ref) |  |  |
| II | 114 | 87 | 0.79 | 0.56-1.11 | 0.167 |
| III | 612 | 270 | 1.45 | 1.16-1.84 | 0.002 |
| IV | 1276 | 322 | 2.79 | 2.23-3.50 | <0.0001 |
| Unknown | 2036 | 424 | 2.02 | 1.58-2.58 | <0.0001 |

| **Supplemental Table 2. Association of Host Characteristics and MCS Scores** | | | | | |
| --- | --- | --- | --- | --- | --- |
| **Variable** | **MCS<50** | **MCS≥50** | **OR** | **95% CI** | **P Value** |
| Age (Continuous) | 3255 | 2450 | 0.98 | 0.98-0.99 | <0.0001 |
| **Sex** |  |  |  |  |  |
| Male | 1631 | 1399 | 1.00 (Ref) |  |  |
| Female | 1624 | 1051 | 1.41 | 1.26-1.59 | <0.0001 |
| **Education** |  |  |  |  |  |
| < High School | 483 | 215 | 1.00 (Ref) |  |  |
| High School/Vocational/AA | 1902 | 1388 | 0.58 | 0.48-0.70 | <0.0001 |
| College Degree | 870 | 847 | 0.50 | 0.41-0.61 | <0.0001 |
| **Alcohol Use** |  |  |  |  |  |
| Never | 1360 | 977 | 1.00 (Ref) |  |  |
| Former | 668 | 400 | 1.20 | 1.02-1.40 | 0.025 |
| Current | 1227 | 1073 | 0.90 | 0.80-1.02 | 0.107 |
| **Smoking Status** |  |  |  |  |  |
| Never | 483 | 505 | 1.00 (Ref) |  |  |
| Former | 2089 | 1563 | 1.40 | 1.19-1.65 | <0.0001 |
| Current | 683 | 382 | 1.69 | 1.38-2.06 | <0.0001 |
| **Past Medical History** |  |  |  |  |  |
| Yes | 2777 | 2036 | 1.00 (Ref) |  |  |
| No | 478 | 414 | 0.77 | 0.66-0.90 | 0.001 |
| **Marital Status** |  |  |  |  |  |
| Married | 2348 | 1842 | 1.00 (Ref) |  |  |
| Widowed | 311 | 251 | 0.91 | 0.75-1.11 | 0.362 |
| Separated | 22 | 8 | 1.84 | 0.80-4.26 | 0.153 |
| Divorced | 359 | 191 | 1.24 | 1.02-1.50 | 0.029 |
| Never Married | 215 | 158 | 0.94 | 0.75-1.18 | 0.606 |
| **Race** |  |  |  |  |  |
| White | 2718 | 2040 | 1.00 (Ref) |  |  |
| Hispanic | 133 | 100 | 0.95 | 0.72-1.26 | 0.726 |
| Black | 258 | 156 | 1.00 | 0.81-1.25 | 0.975 |
| Asian/Pacific Islander | 92 | 109 | 0.72 | 0.54-0.98 | 0.035 |
| Other | 54 | 45 | 0.88 | 0.58-1.33 | 0.552 |
| **Past Treatment** |  |  |  |  |  |
| Yes | 971 | 640 | 1.00 (Ref) |  |  |
| No | 2284 | 1810 | 1.00 | 0.84-1.19 | 0.985 |
| **Histology** |  |  |  |  |  |
| **Non-Small Cell** |  |  |  |  |  |
| Adenocarcinoma | 1547 | 1277 | 1.00 (Ref) |  |  |
| Squamous Cell | 519 | 391 | 1.12 | 0.95-1.31 | 0.173 |
| Large Cell | 111 | 80 | 1.00 | 0.74-1.36 | 0.992 |
| Non-small cell carcinoma – non-specified | 545 | 368 | 1.10 | 0.94-1.29 | 0.244 |
| **Small Cell** | 408 | 216 | 1.32 | 1.09-1.59 | 0.004 |
| **Other** | 125 | 118 | 0.99 | 0.75-1.31 | 0.954 |
| **Stage** |  |  |  |  |  |
| I | 252 | 312 | 1.00 (Ref) |  |  |
| II | 85 | 116 | 0.86 | 0.61-1.20 | 0.361 |
| III | 495 | 387 | 1.55 | 1.24-1.94 | <0.0001 |
| IV | 943 | 655 | 1.76 | 1.43-2.16 | <0.0001 |
| Unknown | 1480 | 980 | 1.81 | 1.45-2.27 | <0.0001 |

| **Supplemental Table 3. Association Between p38 MAPK Variants and PCS Scores** | | | | | | |  |  |  |  |  |  |
| --- | --- | --- | --- | --- | --- | --- | --- | --- | --- | --- | --- | --- |
|  |  | **PCS: Discovery Phase** | |  |  |  |  | **PCS: Validation Phase** | |  |  |  |
| **Gene: SNP** | **Model** | **PCS <50 WW/WV/VV** | **PCS≥50 WW/WV/VV** | **OR** | **95% CI** | **P Value** | **Model** | **PCS <50 WW/WV/VV** | **PCS≥50 WW/WV/VV** | **OR** | **95% CI** | **P Value** |
| *TNFRSF1B:* rs496888 | Dom. | 120/88/14 | 36/51/12 | 0.40 | 0.21-0.75 | 0.004 | Rec. | 117/81/21 | 52/42/7 | 1.81 | 0.66-4.98 | 0.25 |
| *MAPK11:* rs909692 | Dom. | 83/110/28 | 20/62/17 | 0.35 | 0.17-0.73 | 0.005 | Rec. | 79/109/31 | 38/49/14 | 0.85 | 0.37-1.92 | 0.70 |
| *MAP2K6:* rs2716191 | Dom. | 78/91/53 | 22/51/26 | 0.37 | 0.19-0.75 | 0.006 | Rec. | 71/117/31 | 35/47/19 | 0.50 | 0.23-1.08 | 0.077 |
| *MAP2K6:*  rs2715812 | Dom. | 113/93/16 | 36/53/10 | 0.42 | 0.22-0.78 | 0.006 | Add. | 101/89/29 | 61/32/8 | 1.75 | 1.11-2.76 | 0.016 |
| *MAP2K6:*  rs2074028 | Dom. | 94/98/30 | 29/50/20 | 0.41 | 0.21-0.79 | 0.008 | Rec. | 89/101/29 | 44/50/7 | 3.60 | 1.12-11.58 | 0.032 |
| *MAP3K5:*  rs11755484 | Dom. | 190/32/0 | 95/3/1 | 4.85 | 1.50-16.02 | 0.010 | Dom. | 192/26/1 | 87/14/0 | 0.68 | 0.28-1.60 | 0.37 |
| *MEF2B:*  rs12459686 | Rec. | 72/118/32 | 34/43/22 | 0.36 | 0.17-0.78 | 0.010 | Rec. | 91/94/34 | 44/38/19 | 0.69 | 0.32-1.49 | 0.35 |
| *MAP2K6:*  rs989681 | Dom. | 60/100/62 | 18/50/31 | 0.36 | 0.16-0.79 | 0.011 | Dom. | 61/118/40 | 33/46/22 | 1.66 | 0.88-3.12 | 0.12 |
| *MAP2K6:*  rs2716227 | Dom. | 70/98/54 | 19/52/28 | 0.40 | 0.19-0.83 | 0.014 | Dom. | 60/106/53 | 38/47/16 | 1.63 | 0.88-3.01 | 0.12 |
| *MEF2B:*  rs3761081 | Add. | 145/70/7 | 56/35/8 | 0.53 | 0.32-0.88 | 0.014 | Dom. | 162/48/9 | 73/24/4 | 0.95 | 0.49-1.85 | 0.89 |
| *MEF2A:*  rs325381 | Dom. | 178/42/2 | 67/28/4 | 0.42 | 0.21-0.85 | 0.015 | Dom. | 171/46/2 | 74/26/1 | 0.75 | 0.39-1.44 | 0.38 |
| *MAP2K4:* rs1870584 | Dom. | 66/117/39 | 40/43/16 | 2.15 | 1.12-4.12 | 0.022 | Rec. | 82/110/27 | 39/49/13 | 0.87 | 0.37-2.04 | 0.74 |
| *MAP3K5:*  rs9494569 | Add. | 67/118/37 | 37/55/7 | 1.75 | 1.08-2.84 | 0.023 | Add. | 77/106/36 | 41/46/14 | 0.73 | 0.47-1.14 | 0.17 |
| *MAP2K4:*  rs12942507 | Dom. | 93/102/27 | 52/38/9 | 2.05 | 1.10-3.81 | 0.023 | Rec. | 115/88/16 | 49/42/10 | 0.61 | 0.22-1.70 | 0.34 |
| *MAPK14:*  rs13196204 | Add. | 159/56/7 | 64/28/7 | 0.54 | 0.31-0.92 | 0.024 | Rec. | 145/61/13 | 71/29/1 | 5.04 | 0.59-42.7 | 0.14 |
| *TNF:*  rs1800629 | Add. | 174/43/5 | 72/22/5 | 0.51 | 0.28-0.92 | 0.024 | Dom. | 143/70/6 | 75/23/3 | 1.21 | 0.64-2.29 | 0.56 |
| *MAP2K6:*  rs2521365 | Dom. | 114/81/27 | 41/47/11 | 0.51 | 0.28-0.93 | 0.028 | Rec. | 115/90/14 | 47/42/12 | 0.51 | 0.19-1.37 | 0.18 |
| *MEF2D:*  rs1171556 | Dom. | 182/39/1 | 70/25/4 | 0.45 | 0.22-0.92 | 0.029 | Dom. | 174/44/1 | 78/23/01 | 0.76 | 0.38-1.52 | 0.44 |
| *MEF2A:*  rs12593522 | Add. | 167/51/4 | 66/27/6 | 0.54 | 0.31-0.94 | 0.030 | Dom. | 150/63/6 | 68/31/2 | 0.94 | 0.50-1.76 | 0.84 |
| *MAP2K3:*  rs1466314 | Dom. | 128/74/20 | 42/48/9 | 0.52 | 0.29-0.95 | 0.032 | Rec. | 104/99/16 | 42/43/16 | 0.32 | 1.12-0.81 | 0.017 |
| *MAP2K4:*  rs8082185 | Rec. | 116/96/10 | 52/39/8 | 0.23 | 0.06-0.89 | 0.033 | Add. | 113/92/14 | 62/35/4 | 1.40 | 0.85-2.32 | 0.19 |
| *MEF2D:*  rs1750304 | Dom. | 184/38/0 | 70/26/3 | 0.47 | 0.24-0.95 | 0.035 | Dom. | 173/45/1 | 76/25/0 | 0.70 | 0.36-1.37 | 0.30 |
| *TNFRSF1B:* rs17037696 | Rec. | 136/70/16 | 65/33/1 | 9.84 | 1.14-85.33 | 0.038 | Dom. | 141/64/14 | 56/35/10 | 0.66 | 0.37-1.18 | 0.16 |
| *MAPK11:*  rs6010226 | Add. | 75/110/36 | 20/60/19 | 0.62 | 0.40-0.98 | 0.040 | Dom. | 69/117/33 | 38/47/16 | 1.38 | 0.75-2.54 | 0.30 |
| *TNF:*  rs2009658 | Dom. | 148/65/9 | 73/25/1 | 1.99 | 1.02-3.87 | 0.043 | Dom. | 161/51/7 | 69/29/3 | 0.82 | 0.44-1.54 | 0.53 |
| *TNFRSF1B:*  rs1061624 | Rec. | 77/81/64 | 34/48/16 | 2.17 | 1.02-4.61 | 0.043 | Dom. | 71/94/54 | 31/47/23 | 0.80 | 0.43-1.50 | 0.49 |
| *MAPK11:*  rs2076139 | Add. | 124/80/18 | 66/31/2 | 1.71 | 1.02-2.88 | 0.044 | Dom. | 120/93/6 | 61/33/7 | 2.05 | 1.13-3.74 | 0.019 |
| *MAP2K4:* rs9303045 | Add. | 63/111/48 | 26/46/27 | 0.65 | 0.43-0.99 | 0.046 | Dom. | 49/115/55 | 22/55/24 | 0.92 | 0.46-1.83 | 0.81 |
| *MAP2K6:*  rs9302900 | Dom. | 123/83/16 | 65/27/7 | 1.84 | 1.00-3.39 | 0.049 | Dom. | 125/81/13 | 44/48/9 | 0.60 | 0.34-1.08 | 0.090 |
|  | | | |  |  |  |  |  |  |  |  |  |
|  | | | | | | |  |  |  |  |  |  |

| **Supplemental Table 4. Association Between p38 MAPK Variants and MCS Scores** | | | | | | |  |  | |  |  |  |
| --- | --- | --- | --- | --- | --- | --- | --- | --- | --- | --- | --- | --- |
|  |  | **MCS: Discovery Phase** | |  |  |  |  | **MCS: Validation Phase** | |  |  |  |
| ***Gene*: SNP** | **Model** | **MCS <50 WW/WV/VV** | **MCS≥50 WW/WV/VV** | **OR** | **95% CI** | **P Value** | **Model** | **MCS <50 WW/WV/VV** | **MCS≥50 WW/WV/VV** | **OR** | **95% CI** | **P Value** |
| *MAP2K3:* rs1466314 | Dom. | 79/71/19 | 91/51/10 | 2.25 | 1.31-3.87 | 0.003 | Add. | 80/72/14 | 66/70/18 | 0.71 | 0.67-1.08 | 0.11 |
| *TRAF2:* rs3739942 | Rec. | 90/58/21 | 90/58/4 | 5.43 | 1.55-18.95 | 0.008 | Rec. | 93/63/10 | 83/57/14 | 0.84 | 0.29-2.41 | 0.74 |
| *TNFRSF1B:* rs1061628 | Rec. | 58/91/20 | 54/66/32 | 0.41 | 0.20-0.81 | 0.011 | Dom. | 63/68/35 | 48/79/27 | 0.62 | 0.35-1.11 | 0.11 |
| *TNFRSF1A:* rs4149578 | Dom. | 143/25/1 | 117/34/1 | 0.43 | 0.22-0.83 | 0.011 | Dom. | 140/26/0 | 125/29/0 | 0.75 | 0.36-1.57 | 0.45 |
| *MAP2K6A:*  rs2715815 | Add. | 68\82\19 | 47\80\25 | 0.62 | 0.41-0.93 | 0.021 | Dom. | 57/84/25 | 61/63/30 | 1.39 | 0.79-2.43 | 0.25 |
| *MAP2K6:*  rs9302900 | Rec. | 95\59\15 | 93\51\8 | 3.97 | 1.21-13.01 | 0.023 | Rec. | 89/68/9 | 80/61/13 | 0.50 | 0.17-0.49 | 0.21 |
| *MAP2K3:*  rs9899521 | Add. | 111\51\7 | 80\59\12 | 0.60 | 0.39-0.93 | 0.024 | Dom. | 84/75/7 | 91/58/5 | 1.79 | 1.03-3.12 | 0.038 |
| *MAP2K6:*  rs2028049 | Rec. | 76\63\30 | 69\70\13 | 2.61 | 1.13-6.00 | 0.024 | Rec. | 70/76/20 | 66/65/23 | 0.84 | 0.38-1.85 | 0.66 |
| *MEF2A:*  rs10902549 | Dom. | 55\78\36 | 31\82\39 | 0.51 | 0.28-0.94 | 0.031 | Dom. | 41/85/40 | 40/74/40 | 1.23 | 0.64-2.35 | 0.54 |
| *MAP2K6:* rs6501328 | Rec. | 92/62/15 | 84/60/8 | 3.31 | 1.11-9.90 | 0.032 | Add. | 86/67/13 | 83/59/12 | 0.97 | 0.63-1.49 | 0.89 |
| *MAP2K4:* rs12942507 | Dom. | 83/63/23 | 62/77/13 | 0.56 | 0.32-0.96 | 0.034 | Add. | 82/69/15 | 82/61/11 | 1.57 | 1.01-2.43 | 0.046 |
| *TRAF2:* rs10781522 | Rec. | 60/71/38 | 55/82/15 | 2.18 | 1.05-4.54 | 0.037 | Dom. | 63/73/30 | 53/76/25 | 0.76 | 0.43-1.33 | 0.33 |
| *FAS:* rs6586163 | Dom. | 40/87/42 | 25/92/35 | 0.49 | 0.25-0.96 | 0.037 | Dom. | 24/97/45 | 33/67/54 | 2.21 | 1.06-4.63 | 0.035 |
| *MAP2K6:* rs11651488 | Rec. | 98/57/14 | 94/51/7 | 3.15 | 1.07-9.30 | 0.037 | Add. | 92/64/10 | 80/63/11 | 0.80 | 0.51-1.25 | 0.32 |
| *MEF2A:* rs325383 | Rec. | 78/68/23 | 86/59/7 | 2.83 | 1.06-7.55 | 0.038 | Dom. | 69/84/13 | 81/60/13 | 1.79 | 1.03-3.11 | 0.040 |
| *FASLG:* rs10458360 | Rec. | 49/85/35 | 47/85/20 | 2.13 | 1.04-4.38 | 0.039 | Rec. | 48/87/31 | 47/71/36 | 0.83 | 0.43-1.60 | 0.57 |
| *MEF2B:* rs2040562 | Rec. | 86/60/23 | 75/70/7 | 3.06 | 1.05-8.92 | 0.041 | Rec | 62/76/28 | 56/84/14 | 2.61 | 1.11-6.15 | 0.028 |
| *TNFRSF1B*: rs5745984 | Dom. | 156/13/0 | 150/2/0 | 5.34 | 1.05-27.21 | 0.044 | Dom. | 158/8/0 | 151/3/0 | 1.34 | 0.30-5.99 | 0.71 |
| *TNF:* rs2009658 | Rec. | 113/47/9 | 108/43/1 | 9.58 | 1.02-89.75 | 0.048 | Dom. | 114/46/6 | 116/34/4 | 1.44 | 0.79-2.64 | 0.24 |
| *MAP2K4:* rs8064513 | Rec. | 83/69/17 | 71/71/10 | 2.86 | 1.01-8.08 | 0.048 | Rec. | 78/71/17 | 74/66/14 | 0.80 | 0.32-2.00 | 0.63 |
|  | | | |  |  |  |  |  |  |  |  |  |
|  | | | | | | |  |  |  |  |  |  |

| **Supplemental Table 5. Association between Low PCS/MCS Score and Five-Year Overall Survival** | | | | | | | | |
| --- | --- | --- | --- | --- | --- | --- | --- | --- |
| **SF-12 Score** | **Alive** | **Dead** | **Unadjusted HR** | **95% CI** | **P Value** | **Adjusted HR** | **95% CI** | **P Value** |
| **PCS** |  |  |  |  |  |  |  |  |
| High | 645 | 822 | 1.00 (Ref) |  |  | 1.00 (Ref) |  |  |
| Low | 1,344 | 3,579 | 1.91 | 1.77-2.06 | <0.001 | 1.63 | 1.51-1.77 | <0.001 |
| **MCS** |  |  |  |  |  |  |  |  |
| High | 956 | 1,787 | 1.00 (Ref) |  |  | 1.00 (Ref) |  |  |
| Low | 1,033 | 2,644 | 1.34 | 1.26-1.43 | <0.001 | 1.23 | 1.16-1.32 | <0.001 |
|  | | | |  |  |  |  |  |
|  | | | | | | | | |

| **Supplemental Table 6. Association Between Genetic Variants in the p38 MAPK Pathway and 5-Year Overall Survival** | | | | | | |  |  |  |  |  |  |
| --- | --- | --- | --- | --- | --- | --- | --- | --- | --- | --- | --- | --- |
| **Discovery** | | | | | | | **Validation** | | | | | |
|  | **Model** | **Dead WW/WV/VV** | **Alive WW/WV/VV** | **HR** | **95% CI** | **P Value** | **Model** | **Dead WW/WV/VV** | **Alive WW/WV/VV** | **HR** | **95% CI** | **P Value** |
| *MAP3K5:* rs3765259 | Dom. | 66/111/59 | 17/46/22 | 0.56 | 0.40-0.79 | 0.0008 | Dom. | 51/121/52 | 25/36/35 | 1.40 | 0.98-2.00 | 0.063 |
| *MAP2K6:* rs817545 | Rec. | 55/116/65 | 27/41/17 | 1.70 | 1.23-2.35 | 0.001 | Dom. | 54/109/61 | 17/58/21 | 0.83 | 0.58-1.17 | 0.29 |
| *MAP2K6:* rs4968791 | Rec. | 65/107/59 | 29/43/13 | 1.68 | 1.20-2.35 | 0.003 | Rec. | 56/116/50 | 22/52/20 | 1.19 | 0.82-1.72 | 0.37 |
| *MAPK11:* rs909692 | Rec. | 75/125/35 | 28/47/10 | 1.82 | 1.21-2.73 | 0.004 | Rec. | 80/111/33 | 37/47/12 | 0.73 | 0.47-1.14 | 0.17 |
| *MAP2K6*: rs2716195 | Dom. | 173/57/6 | 55/26/4 | 0.62 | 0.44-0.88 | 0.006 | Dom | 163/53/8 | 69/25/2 | 1.16 | 0.82-1.63 | 0.41 |
| *TNFRSF1B:* rs7552664 | Rec. | 144/80/12 | 47/28/10 | 0.39 | 0.19-0.80 | 0.011 | Rec. | 125/85/14 | 50/41/5 | 1.38 | 0.75-2.53 | 0.31 |
| *MAP2K6:* rs6501328 | Dom. | 131/86/19 | 45/36/4 | 0.69 | 0.51-0.93 | 0.016 | Rec. | 122/84/18 | 47/41/7 | 1.61 | 0.96-2.72 | 0.086 |
| *MEF2B:* rs12609573 | Add. | 68/116/52 | 33/38/14 | 1.28 | 1.03-1.58 | 0.023 | Dom. | 59/115/50 | 24/49/23 | 0.79 | 0.57-1.10 | 0.16 |
| *MAPK11*: rs6010226 | Add. | 74/117/44 | 21/53/11 | 1.29 | 1.03-1.61 | 0.026 | Rec. | 74/116/34 | 33/48/15 | 0.70 | 0.45-1.08 | 0.10 |
| *MEF2B:* rs12459686 | Add. | 87/115/34 | 19/46/20 | 0.79 | 0.63-0.98 | 0.032 | Dom. | 94/91/39 | 41/41/14 | 0.85 | 0.63-1.14 | 0.27 |
| *MAP2K6*: rs2715825 | Rec. | 105/110/21 | 30/40/15 | 0.53 | 0.30-0.95 | 0.033 | Add. | 107/97/20 | 51/36/9 | 1.16 | 0.92-1.47 | 0.21 |
| *MEF2D:* rs16837415 | Dom. | 205/30/1 | 79/6/0 | 1.69 | 1.04-2.73 | 0.034 | Dom. | 180/43/1 | 76/20/0 | 0.87 | 0.60-1.28 | 0.49 |
| *MAPK14:* rs851006 | Dom. | 136/82/18 | 48/32/5 | 0.72 | 0.53-0.98 | 0.034 | Rec. | 131/79/14 | 58/32/6 | 1.27 | 0.71-2.28 | 0.42 |
| *MAP2K4:* rs4791490 | Rec. | 145/86/5 | 48/30/7 | 0.22 | 0.05-0.92 | 0.039 | Dom. | 138/78/8 | 62/28/6 | 1.18 | 0.85-1.63 | 0.32 |
| *MEF2D:* rs10159180 | Dom. | 62/123/51 | 33/34/18 | 1.44 | 1.01-2.05 | 0.041 | Rec. | 53/111/60 | 24/50/22 | 1.48 | 1.05-2.09 | 0.026 |
| *TNF:* rs3093662 | Dom. | 203/30/3 | 79/6/0 | 1.54 | 1.01-2.35 | 0.046 | Dom. | 199/23/2 | 79/16/1 | 0.72 | 0.44-1.18 | 0.20 |
| *MEF2A*: rs325383 | Rec. | 122/95/19 | 42/32/11 | 0.58 | 0.34-0.99 | 0.046 | Dom. | 115/91/18 | 35/53/8 | 0.84 | 0.62-1.13 | 0.25 |
| *MEF2D:* rs16837408 | Dom. | 179/47/10 | 60/23/2 | 0.70 | 0.49-0.99 | 0.047 | Dom. | 174/43/7 | 77/19/0 | 1.31 | 0.93-1.87 | 0.13 |
|  | | | | | | |  |  |  |  |  |  |
|  | | | | | | |  |  |  |  |  |  |
